# Chromium Bioaccumulation and Its Impacts on Plants: An Overview

**DOI:** 10.3390/plants9010100

**Published:** 2020-01-13

**Authors:** Anket Sharma, Dhriti Kapoor, Junfeng Wang, Babar Shahzad, Vinod Kumar, Aditi Shreeya Bali, Shivam Jasrotia, Bingsong Zheng, Huwei Yuan, Daoliang Yan

**Affiliations:** 1 State Key Laboratory of Subtropical Silviculture, Zhejiang A&F University, Hangzhou 311300, China; 2 School of Bioengineering & Biosciences, Lovely Professional University, Punjab 144411, India; 3 School of Land and Food, University of Tasmania, Hobart, Tasmania 7005, Australia; 4 State Higher Education Department, Jammu and Kashmir 180001, India; 5 Mehr Chand Mahajan D.A.V. College for Women, Chandigarh 160036, India; 6 Department of Zoology, Guru Nanak Dev University, Amritsar 143005, India

**Keywords:** heavy metal, reactive oxygen species, oxidative burst, antioxidants

## Abstract

Chromium (Cr) is an element naturally occurring in rocky soils and volcanic dust. It has been classified as a carcinogen agent according to the International Agency for Research on Cancer. Therefore, this metal needs an accurate understanding and thorough investigation in soil–plant systems. Due to its high solubility, Cr (VI) is regarded as a hazardous ion, which contaminates groundwater and can be transferred through the food chain. Cr also negatively impacts the growth of plants by impairing their essential metabolic processes. The toxic effects of Cr are correlated with the generation of reactive oxygen species (ROS), which cause oxidative stress in plants. The current review summarizes the understanding of Cr toxicity in plants via discussing the possible mechanisms involved in its uptake, translocation and sub-cellular distribution, along with its interference with the other plant metabolic processes such as chlorophyll biosynthesis, photosynthesis and plant defensive system.

## 1. Introduction

Chromium (Cr) is a silver-colored hard metal naturally occurring in rocky soils, and volcanic dust. Chromium is the 24th element having a molecular weight of 51.1 a.m.u. and a density of 7.19 g/cm^3^ [1]. The Agency for Toxic Substances and Disease Registry [2] has ranked Cr the 17th among the most hazardous substances. It has been classified as the number one carcinogen according to the International Agency for Research on Cancer [3]. Therefore, this metal needs an accurate understanding of its uptake, transport and bioaccumulation within plants and a thorough investigation in soil–plant systems.

Chromium can easily convert from one oxidation state to another due to its high redox potential and intricate electronic and valence shell chemistry [4,5]. It exists in a wide range of oxidation states, but the most common and stable states are Cr (VI) “hexavalent” and Cr (III) “trivalent” [6]. Both forms of Cr vary significantly with respect to their bioavailability in soil, translocation and toxicity within plants [4]. The Cr (III) occurs in the form of chromite (FeOCr_2_O_3_), whereas Cr (VI) occurs in association with oxygen to form chromate (CrO_4_^2−^) or dichromate (Cr_2_O_7_^2−^), that are highly toxic to living organisms [7]. Based on its activity, former is the most stable form of Cr, whereas the highest noxious one for plants is the Cr (VI). Under physiological conditions, Cr (VI) enters the cells and may get reduced to Cr (V), Cr (IV), thiylradicals, hydroxyl radicals and finally Cr (III). All these oxidation states disrupt the cellular integrity of cells by attacking proteins, DNA and membrane lipids [8,9].

Hexavalent Cr (VI) is used in several industrial applications such as electroplating, dyeing of textiles, leather processing, steel production and tanning industry, resulting in discharge of chromium-containing effluents. This ultimately causes significant elevation in Cr contents in the environment [10]. Due to its high solubility in water and soil, Cr (VI) is regarded as a hazardous ion that contaminates groundwater and can be transferred through the food chain [10,11,12]. It also occurs in air, water and soil at different concentrations. Its contents in freshwater and seawater vary from 0.1 to 117 μg L^−1^ and 0.5 to 50 μg L^−1^ respectively. The weathering of Cr containing rocks and leaching of soils discharge significant Cr contents into the aquatic environment [7]. Cr contents in soil ranges between 10 and 50 mg kg^−1^ under natural conditions, however, its concentration in agricultural soils can reach up to 350 mg kg^−1^ of the soil [7]. The United States Environmental Protection Agency (USEPA) [13] has listed Cr among the 14 most dangerous substances that can cause serious health issues in living organisms. Cr can have both beneficial and harmful effects on human health depending on its uptake, exposure time and oxidation state. The trivalent form of Cr (III) is an important nutrient for humans and according to the World Health Organization [14], its daily ideal intake is between 50 and 200 μg day^−1^ for the metabolism of carbohydrates, proteins and fatty acids. However, its excess in the body poses serious health concerns. Moreover, hexavalent Cr (VI) is 10–100 folds more harmful than Cr (III), which can cause allergies and skin problems.

To date, Cr does not have any known biological role in plant physiology [15]. It is generally perceived that excessive Cr levels in plant tissues may provoke several morpho-physiological and biochemical processes in plants [16,17]. Any metal toxicity is attributed to a complex series of metal interactions with the genetic processes, signal transduction and pathways and cellular macromolecules [18,19,20]. Hence, Cr toxicity is reported to affect plant growth and impedes their essential metabolic processes [21]. Typically, Cr toxicity reduces plant growth by inducing ultrastructural modifications of the cell membrane and chloroplast, persuading chlorosis in the leaves, damaging root cells, reducing pigment content, disturbing water relations and mineral nutrition, affecting transpiration and nitrogen assimilation and by altering different enzymatic activities [15,22,23,24,25]. All these toxic effects of Cr might be due to the over production of reactive oxygen species (ROS), which ultimately disrupt the redox balance in plants [25]. Taking all into consideration, we review the literature that addresses Cr uptake, translocation and sub-cellular distribution in plants. We also discuss different effects of Cr on plant pigments, photosynthetic parameters, enzymatic and non-enzymatic antioxidative system and various endogenous levels of plant hormones (Table 1).

## 2. Chromium Uptake, Translocation and Sub-Cellular Distribution

Plant roots secrete various organic acids such as citrate and malate that modify the solubility of metals present in insoluble form in the soil by acting as ligands [42,43,44,45]. Srivastava et al. [46] had shown an increased accumulation of Cr in tomato plants due to the presence of citrate, aspartate and oxalate, which converted inorganic Cr into organic complexes, which are readily available for the plant to uptake. Chromium appears to have no essential role in plant metabolism, hence, there is no specific mechanism for its uptake in plants [27]. Skeffington et al. [47] had proposed a mechanism for uptake of both Cr (III) and Cr (VI) in barley (*Hordeum vulgare*) plants. Nonetheless, specific carriers responsible for the absorption of essential ions also aid in the uptake of Cr [4]. The uptake of Cr (III) in plants undergoes through passive mechanism [48], however, Cr (VI) is uptaken through the plasma membrane, which is an active process involving carriers of essential anions such as sulfate [49,50]. Further, due to the structural similarity of Cr (VI) with phosphate and sulfate, its uptake by root cells involves phosphate or sulfate transporters [51,52].

The distribution and translocation of Cr within plants depend upon the plant species, the oxidation state of the Cr ions, and also its concentration in the growth medium [4]. Compared to other heavy metals, the mobility of Cr in the plant roots is low. Therefore, the concentration of Cr in the roots is sometimes 100 times higher than in the shoots [48,53]. For instance, Cr concentration was observed to be highest in the cytoplasm and intercellular spaces of rhizome and root cell wall of *Iris pseudacorus* [54]. The higher accumulation of Cr in roots might be attributed to the sequestration of Cr in the vacuoles of root cells as a protective mechanism [55]. Thus, this mechanism provides some natural tolerance to plants towards Cr toxicity [40]. Furthermore, the translocation of Cr from the roots to the aerial shoots is very limited and it depends on the chemical form of Cr inside the tissue [4]. In plant tissues, the Cr (VI) is converted to Cr (III) that has the tendency to bind to the cell walls, which hinders the further transport of Cr within plant tissues [56].

Numerous metal transporter gene families including CDF (cation diffusion facilitator), HMA (heavy metal ATPase), ATP binding cassette (ABC) superfamily and ZIP (ZRT, IRT-like protein) have been identified for different metals like Pb, Cd, Zn and As [4,57,58,59,60,61]. However, the role of transporter families in the translocation of Cr in plants is still unclear. The translocation of Cr (VI) to shoots is an active process that involves phosphate and sulfate transporters [4]. Hence, the translocation of Cr might be mediated by iron (Fe) and sulfur (S) channels in the roots that lead to the competition between metals e.g., Fe and Cr [4,62]. Cary et al. [63] had reported Cr uptake and translocation to the aerial shoots in Fe hyperaccumulators *Brassica rapa* and *Spinacia oleracea* signifying that Cr may be transported through Fe channels. However, the presence of Fe in the growth media reduced Cr translocation to the shoots [64], which could be due to the competition of carrier channels or due to the precipitation of Fe with Cr.

## 3. Effect of Cr on Nutrient Uptake

Heavy metal stress affects nutrient uptake in plants by interacting with other essential minerals. Chromium restricts the uptake of nutrients in soil by forming insoluble compounds [65]. Nutrient uptake is thereby inhibited by the metal toxicity especially when the concentration of the metal exceeds its permissible limits [66]. For instance, excessive Cr had been observed to reduce the uptake of essential minerals like iron (Fe), magnesium (Mg), phosphorus (P) and calcium (Ca) by masking the sorption sites and forming insoluble complexes [56,66]. However, Cr transport to different parts of *Citrullus* plants had increased leading to enhancement in the concentrations of manganese (Mn) and P, and reduction in sulphur (S), copper (Cu), zinc (Zn) and iron (Fe) contents in the leaves, suggesting that Cr disturbs the nutrient balance [67]. Turner and Rust [68] also suggested the similar effects of Cr on the uptake of various nutrients under Cr toxicity. A gradual decrease in the uptake of micronutrients like Zn, Cu, Fe, Mn and macronutrients like potassium (K), P and nitrogen (N) had been noticed in the paddy plants (*Oryza sativa* L.) under excessive Cr exposure [69]. This reduced nutrient uptake may occur due to decline in the root growth and impairment of the root penetration under Cr toxicity, or may be due to the decrease in essential element translocation because of the displacement of nutrients from the physiologically important binding sites [70,71].

## 4. Effect of Cr on Chlorophyll Molecules and Photosynthetic Performance

Foliar content of chlorophyll pigments including total chlorophyll, chlorophyll a (Chl a) and chlorophyll b (Chl b) were assayed under Cr treatment, which showed significant decrease in pigment accumulation of *Catharanthus roseus* plants [72]. This could be due to the inhibition of chlorophyll biosynthesis under Cr stress [73,74]. Increased concentration of Cr may lead to the deterioration of the chlorophyll content in many plants [75]. Plants exposed to Cr stress showed depleted chlorophyll contents that might be due to the disrupted chlorophyll biosynthesis [76]. Interestingly, an enzyme involved in chlorophyll biosynthesis, i.e., δ-aminolevulinic acid dehydratase (ALAD) is being inhibited by Cr due to the impairment in utilizing the δ-aminolevulinic acid [77].

Effect of Cr was also depicted on pigment contents viz. chlorophyll of vetiver [78] where alterations in the photosynthetic pigments were observed. Cr induced toxicity had been reported to decrease the chlorophyll contents in different plants species such as *Pistia stratiotes* [79], *Citrus limonia* and *Citrus reshni* [80], *Zea mays* [81,82], *Hibiscus esculantus* [83], *Camellia sinensis* [84], *Glycine max* [85] and *Ocimum tenuiflorum* [31]. Decrease in chlorophyll contents under Cr toxicity could be due to the impairment of chlorophyll biosynthesis enzymes, which are compromised under Cr toxicity [86,87]. Degradation of ALAD could occur under Cr toxicity leading to a decrease in chlorophyll level [88]. Hence, photosynthetic capacity of plants is compromised under Cr stress due to interaction with biosynthesis of chlorophyll molecules by inhibiting vital enzymes contributing in photosynthesis. Excessive Cr affects photosynthetic system by targeting the Calvin cycle enzymes, photosynthetic electron transport and thylakoid membrane [89]. Therefore, gradual decrease in the net photosynthetic rate can be observed in the plants treated with higher concentration of Cr [90].

Changes in the level of photosynthetic pigments give an important information regarding the toxic effects of heavy metals, e.g., Cr, Ni, Pb and Cd [70,74,91,92,93,94]. However, reduced chlorophyll contents may be observed due to the increased activity of enzymes like chlorophyllase and deficiency of nutrients, i.e., because of the translocation of the metals to shoots in higher concentration [91,95]. Moreover, a significant decrease in transpiration rate, net photosynthetic rate, intercellular CO_2_ concentration and stomatal conductance in the leaves were observed where Cr toxicity reduced these parameters by 71%, 36%, 25% and 57% respectively [96]. Cr also poses hazardous effects on gas exchange as shown by the multiple linear regression (MLR) analysis that expressed negative β-regression coefficients for all the parameters of gas exchange [97]. Davies Jr et al. [96] had noticed that Cr inhibited photosynthetic process by targeting photosystem II (PSII). Hence, chlorophyll fluorescence seems quite useful tool to study photosynthetic apparatus and action of PSII under heavy metal stress.

## 5. Reactive Oxygen Species (ROS) and Oxidative Stress

A rather common and frequent effect of heavy metal stress is the overproduction of ROS including hydroxyl radicals (OH^−^), hydroperoxyl radicals (HOO), superoxide (O_2_^−^), the peroxinitrite (OONOˉ) ion, the paramagnetic singlet oxygen (^1^O_2_), nitrogen oxide radical (NO), hydrogen peroxide (H_2_O_2_), ozone (O_3_) and hypochlorous acid (HOCl) molecules [98,99,100,101]. This process is considered as one of the primary cause for the alterations in plant biology at biochemical level under heavy metal toxicity [70,73,74,102,103,104,105,106]. Plants may suffer through various drastic physiological changes, which are mainly due to the imbalance in the generation and scavenging of ROS, termed as an oxidative burst [70,74,94,102,104,107,108,109,110,111,112]. Heavy metals like copper (Cu), nickel (Ni), cadmium (Cd), Cr and arsenic (As) have the tendency to generate ROS if they exceed permissible limits [70,113,114,115].

On the basis of physical and biochemical characteristics of bioactive-metals, these metals can be classified into two groups; redox metals like Cr, Cu, Fe and non-redox metals like Cd, Hg, Ni, Zn, etc. Redox active metals have the capacity to produce oxidative injuries in plants via Haber–Weiss and Fenton reactions, that consequently generate ROS and leading to disturb the balance between prooxidant and antioxidant level [116]. However, redox-inactive metals form covalent bonds with the protein sulfhydryl groups as these metals have the tendency of sharing the electrons.

**Figure 1:**
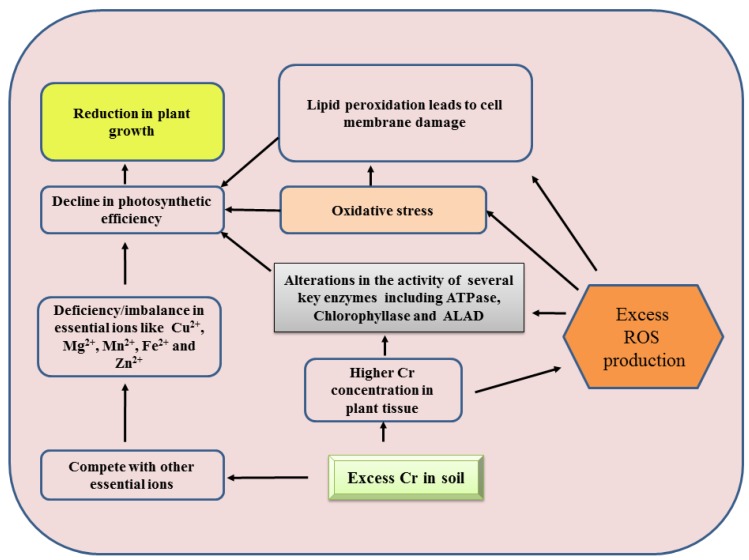
Consequences of oxidative stress generated under chromium toxicity.

When Cr metal interacts with the proteins at its catalytic site or any other site, it deactivates the active sites of enzymes by binding specific functional groups of proteins, thus leading to the alteration of enzymatic activities [117,118]. Furthermore, dislocation of critical cations from the specific enzyme binding sites disturbs the equilibrium of ROS in cells, and as a consequence ROS are generated in drastic amount [119]. Chromium metal has also tendency to bind and utilize the reduced form of glutathione (GSH) and its derivatives, which plays a significant role in ameliorating these ROS [120]. Besides, NADPH oxidase (present on plasma membrane) also leads to oxidative stress as they are linked with the Cr [121,122,123]. In the presence of Cr metal, the NADPH oxidases may consume cytosolic NADPH and produce free radical O_2_^−^, which is rapidly converted to H_2_O_2_ by superoxide dismutase enzyme (SOD) [124]. Free radicals generated by Cr in association with NADPH oxidase remains outside the plasma membrane, where pH remains usually low in comparison to inner side of the cell [125]. Reports suggest that the enhanced generation of ROS in plants under Cr toxicity leads to oxidative burst by causing damage to DNA, lipids, pigments, proteins and stimulates the process of lipid peroxidation (Figure 1) [126,127]. Carrier membrane stimulates the absorption of Cr and over productions of ROS further influences the plasma membrane [41].

There are several reports documented where drastic increase in ROS was observed [22,74,104,128] with increased malondialdehyde (MDA) contents under Cr toxicity [129]. Alterations in different physiological and biochemical activities have been observed in *Triticum aestivum*, *Vallisneria spiralis* and *Ocimum tenuiflorum* [22,31,88] where Cr metal stimulated the deterioration of membrane permeability by generating MDA. Similarly, increased levels of MDA were found in both roots and leaves of *Kandelia candel* (L.) plants in a dose dependent manner suggesting its gradual uptake under a timely manner [30].

## 6. Effect of Cr on Enzymatic Antioxidative System

Activities of various antioxidative enzymes drastically change in plants when subject to Cr toxicity. ROS leads to oxidative stress, which may affect different subcellular compartments sensitive to ROS. Superoxide dismutase (SOD) is considered as a first line of defense against various stresses in almost all the aerobic organisms [70,130]. Dismutation of superoxide ion is catalyzed by the SOD enzyme, which is localized in almost all the cellular compartments, leading to the production of hydrogen peroxide and release of oxygen [130]. SOD is involved in the Asada–Halliwell cycle in chloroplasts and also present in cytosol, apoplasts, mitochondria and peroxisomes [131]. For the removal of ROS, catalase (CAT) enzyme also plays crucial role, hence considered as important antioxidant enzyme [132]. Dismutation of H_2_O_2_ into O_2_ and H_2_O is undergone by this enzyme [133].

When exposed to Cr (III) stress for seven days, chamomile plants showed increased accumulation of Cr mainly in the roots of the plants, which contained high concentrations of ROS, nitric oxide and thiols. At higher concentration of Cr (III), SOD activity specifically was increased in the roots, while level of H_2_O_2_ showed irregular trend under different concentrations of Cr due to the altered activities of various peroxidases [28]. Different concentrations of Cr (VI) (50, 100, 200 and 300 µM L^−1^) escalated the production of H_2_O_2_ leading to the lipid peroxidation and triggered the activities of antioxidative enzymes like SOD and guaiacol peroxidase (GPX) in comparison to control plants [41]. In contrast, activities of peroxidase (POD), SOD and CAT were decreased when subjected to Cr (III) in a dose dependent manner [26].

Maintaining metabolic functions under stress conditions is crucial for plants to survive. Therefore, a balance between generation and scavenging of ROS is required, which is achieved by regulating the production of enzymatic and non-enzymatic antioxidants [134]. Hence, the ability of plants to cope with oxidative stress is characterized by the degree of antioxidant activities [102,104]. However, activities of these antioxidants may vary with the duration, crop species and tissues under any stress condition [135]. For instance, *Echinochloa colona* plants showed increased activities of POD and CAT in tolerant calluses in comparison to non-tolerant ones [29]. At 0.5 mg L^−1^ concentration of Cr (VI), CAT activity was increased, however decreased at higher concentrations (1.0–2.0 mg L^−1^). Activity of CAT was measured highest at 2.0 mg L^−1^ concentration of Cr in the roots of *K. candel* but decreased at higher concentration [30]. As CAT is an iron-porphyrine biomolecule, reduction in CAT activity indicates that Cr has the potential to interact with iron in metabolic pool or it may influence the presence of active form of iron [136]. Cr toxicity has detrimental effects on antioxidant enzymes such as POD, GPX, glutathione reductase (GR) and ascorbate peroxidase (APX), hence resulting in the inhibition of enzyme activities [129,137]. Plants equipped with an efficient antioxidant system are more capable to withstand and tolerate higher Cr concentrations. Failure to do so results in the breakdown of the plant defense system hence activities of antioxidants are jeopardized leading to reduced plant growth or even leading to plant death.

## 7. Effect of Cr on Non-Enzymatic Antioxidative System

Apart from the enzymatic antioxidants, plants are also comprised of a complex non-enzymatic antioxidant defense system to avoid the toxic effects of ROS. These non-enzymatic antioxidants consist of low molecular weight molecules such as ascorbic acid, glutathione (GSH), phenolic acids, carotenoids, flavonoids, etc. [94,138] and some high molecular weight secondary metabolites such as tannins [139]. Biosynthesis and accumulation of these non-enzymatic antioxidants by plants could be due to two main reasons. First, plants have an innate ability to synthesize a variety of phytochemicals to carry out their normal physiological functioning or to protect them from any pathogenic or herbivores. Second, plants also synthesize phytochemicals to respond to the environmental factors which could be due to their natural tendency of defense against any biotic and abiotic stress [140]. Therefore, these lower molecular weight antioxidants are synthesized and act as a redox buffer to interact with cellular components and directly influence plant growth and development by modulating different processes from mitosis to cell elongation and to senescence. Hence, it is crucial for plants to synthesize these antioxidants under stressed conditions. Glutathione is a redoxactive molecule that can be present in a reduced form GSH or an oxidized form GSSG. It plays important roles in the plant defensive system including biosynthetic pathways, detoxification, antioxidant biochemistry and redox homeostasis [141,142]. GSH acts as an antioxidant by quenching ROS and is involved in the ascorbate-glutathione cycle, which eliminates damaging peroxides [143]. In poplar trees, glutathione (GSH) biosynthesis was stimulated under Cr toxicity [144]. In the leaf extracts of tomato (*Lycopersicon esculentum*), maize (*Zea mays*) and cauliflower (*Brassica oleracea*) plants, GSH level increased subjected to Cr toxicity [145]. Alterations were observed in the glutathione pool dynamics where individual level of GSSG and GSH and GSH/GSSG ratio was affected, however sorghum (*Sorghum biclor*) plants showed potential to scavenge the free radicals generated under Cr toxicity [146].

Under sub-optimal conditions, level of antioxidants may decrease or increase depending on the severity of stress. For instance, a sharp decline in the GSH pool was observed under Cr stress, which severely affected the roots of the plants. Many reports suggested the oxidation of various cellular thiols like GSH and cysteine in plants subjected to Cr (VI) stress in in vitro conditions [147]. Therefore, to maintain the redox homeostasis of the cell and for scavenging of free radicals, the interconversion of reduced and oxidized forms of glutathione (GSH and GSSG respectively) is required.

A non-enzymatic antioxidant “carotenoid” contributes in providing protection to the chlorophylls against stress conditions by replacing peroxides and scavenging of photodynamic reactions [148]. For instance, carotenoid level increased in *Capsicum annuum* plants subjected to industrial effluents specifically containing Cr [27], acting as a defensive mechanism for capsicum plants to scavenge the free radicals. Other non-enzymatic antioxidants such as cysteine, proline, nonprotein thiol, etc. may also contribute in modulating resistance against Cr toxicity and protect the macromolecules from the free radicals generated during the oxidative burst [77]. In one of the reports, carotenoids, non-protein thiol (NP-SH) and cysteine level increased in the plants subjected to Cr [72].

For providing protection against different types of stresses, accumulation of compatible osmolytes like proline (Pro) occurs in most of the plants for providing membrane stability and osmotic adjustment [94,149]. Proline contents can increase against different types of biotic and abiotic stresses such as salinity, drought, temperature, heavy metal and pathogen attack [77]. For instance, contents of Pro increased in *Ocimum tenuiflorum* L. under Cr stress, which acted as an antioxidant by providing protection against the hazardous effects of metal [31]. Reports suggested that proline is the only amino acid that accumulates in the leaves of plants under stress conditions [150]. It starts accumulating even at low doses of stress and increases in a dose dependent manner. Hence, accumulation of proline contributes to osmotic adjustment when it gets accumulated in tissues and acts as a dependent marker for genotypes for the stress tolerance [150]. Polyamines are other non-enzymatic osmoprotectants, which increase under different abiotic stress conditions [151], and also associated with the boosting up of plant defensive mechanisms [92,152]. Polyamines including putrescine, spermine and spermidine have been investigated to increase Cr tolerance in plants under Cr toxicity [38].

## 8. Effect of Cr on the Endogenous Levels of Plant Hormones

Plant hormones control and regulate plant growth and development through different biochemical and physiological process. These hormones may act either close to or remote from their synthesis site to regulate responses to environmental stimuli or genetically programmed developmental changes [153]. Hormones thus have a vital role in plant adaptation to abiotic stress, from which the plant may attempt to escape or survive under stressful conditions [154]. Thus, abiotic stresses often alter the production, distribution or signal transduction of growth as well as stress hormones, which may promote specific protective mechanisms. The phytohormone abscisic acid (ABA) plays a vital role against abiotic stresses, thus it is considered as a stress hormone. Under different types of abiotic stress conditions, endogenous level of ABA are increased drastically in plants, which boosts up the signaling pathways and activates expression of ABA-responsive genes [155]. For instance, strong expression of ABA biosynthesis genes (*OsNCED2* and *OsNCED3*) and four ABA signaling genes were upregulated in response to heavy metal stress [156]. Salicylic acid (SA) also provides protection under Cr stress. SA plays a significant positive role in growth and development of plants as well as ripening and abiotic stress tolerance [157]. Interaction of ABA and SA also contributes in boosting the defensive strategies of plants against Cr toxicity [158]. Apart from this, SA also plays essential role in combination with jasmonic acid (JA) and ethylene, where the interaction of these hormones provides tolerance against Cr stress [159,160]. The biosynthesis, transportation and accumulation of these plant hormones boost the signaling pathways, activating certain antioxidant gene expressions and stimulating the production of osmoprotectants such as proline, soluble sugars, amino acids, etc. [161].

Indole acetic acid (IAA), a plant hormone of auxins contributes to the growth and development of plants under ideal as well as stressed conditions [162]. IAA plays a key role in plant adaptation to heavy metal stress by either increasing the membrane permeability or by increasing the concentration of osmotically active solutes [16,163]. The level of IAA usually increases in the plants under metal toxicity by affecting different metabolic activities of plants including growth and hormonal balance [164]. Gibberellins (GAs) are considered as comprehensive class of tetracyclic diterpenoid carboxylic acid compounds. Different forms of GAs have potential to play the role of growth hormone in higher plants such as GA1 and GA3 [165]. Generally, at low concentration of metal like Cr leads to the elevated level of GA3, although its high concentration reduces the GA3 content [166]. Apart from these, plant hormone like cytokinins (CKs), contributes in the regulation of plant development by stimulating cell division and elongation. Cr stress alters endogenous level of CKs suggesting that CKs are also involved in tolerating the stress [155]. A few reports also indicated the reduction in the synthesis of CKs and their transport from roots to other aerial parts of plants during Cr toxicity and they are also found to interact with other plant hormones [167].

## 9. Conclusions and Prospects

This review illustrates an overview of Cr metal effects on plant growth and development. Plants uptake Cr via roots, which causes nutrient imbalance, root injury as well as leaf chlorosis. Cr toxicity also targets chlorophyll biosynthesis by inhibiting the activity of vital enzymes. Additionally, it also results in oxidative stress by targeting cellular membranes and biomolecules resulting in retarded plant growth, induction of chlorosis and wilting of leaves. Although a handful of data is available that provides useful information to understand chromium interaction with other essential metal ions. The mechanisms generating Cr-induced toxicity at the protein and molecular level still need to be explored in detail. Furthermore, exploration of Cr tolerance mechanism and homeostasis are essential for sustainable crop production, which is poorly understood in many ecosystems. Therefore, it is essential to understand the possible means to reduce Cr uptake and its negative impacts on environment especially in plants.

## Figures and Tables

**Table 1:** Effects of chromium metal on different physiological processes in plants.

Plant Species	Physiological Response	Reference
*Camellia sinensis*	Increased SOD and CAT activities	Tang et al. [26]
*Capsicum annuum*	Increased carotenoid content	Oliveira [27]
*Chamomilla recutita*	Increased MDA level	Kováčik et al. [28]
*Echinochloa colona*	Increased CAT and POD activities	Samantaray et al. [29]
*Kandelia candel*	Increased MDA content, and activities of CAT and SOD	Rahman et al. [30]
*Ocimum tenuiflorum*	Increased proline level	Rai et al. [31]
*Oryza sativa*	Increased POD activity	Ma et al. [32]
*Oryza sativa*	Increased ethylene synthesis	Trinh et al. [33]
*Oryza sativa*	Increased CAT and SOD activities	Zhang et al. [34]
*Oryza sativa*	Increased POD activity	Xu et al. [35]
*Phaseolus vulgaris*	Decreased carotenoids	Aldoobie and Beltagi [36]
*Pisum sativum*	Decreased APX activity	Duhan [37]
*Pterogyne nitens*	Increased spermidine level	Paiva et al. [38]
*Raphanus sativus*	Increased glycine-betaine content	Choudhary et al. [39]
*Triticum aestivum*	Increased MDA contents	Ali et al. [22]
*Triticum aestivum*	Increased lipid peroxidation	Zhang et al. [34]
*Vigna radiata*	Decreased glutathione level	Shanker et al. [40]
*Zea mays*	Increased SOD and GPX activities	Maiti et al. [41]
*Zea mays*	Increased lipid peroxidation and H_2_O_2_ content	Maiti et al. [41]

Abbreviations: Malondialdehyde—MDA; Superoxide dismutase—SOD; Catalase—CAT; Ascorbate peroxidase—APX; Peroxidase—POD; Guaiacol peroxidase—GPX; Hydrogen peroxide—H_2_O_2._
